# Supplementary material for: Glucocorticoid-Induced Leucine Zipper Protein and Yeast-Extracted Compound Alleviate Colitis and Reduce Fungal Dysbiosis
Source: Biomolecules. 2024 Oct 17;14(10):1321. doi: 10.3390/biom14101321 (PMC11506796; doi:10.3390/biom14101321)
Supplement: Supplementary file 1 [file biomolecules-14-01321-s001.zip › biomolecules-3238829-supplementary.pdf]

Supplementary figures

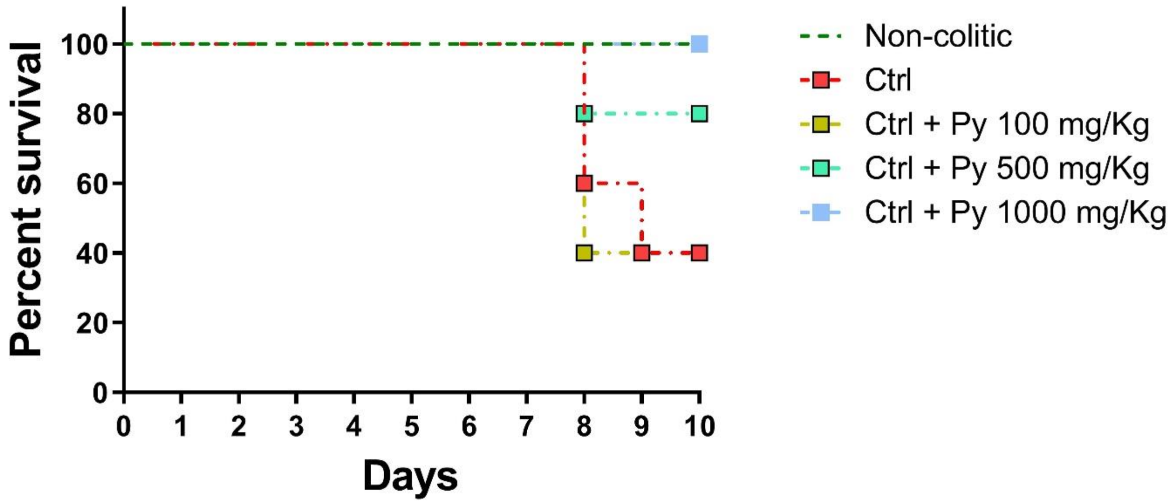

Figure S1. Dose-dependent protective effect of Py o DSS-associated mortality. Kaplan-Meier curve depicting mortality in mice with DSS-induced colitis (3%, 5 days). Py was administered orally at a dose of 100, 500, or 1000 mg/kg, from day 0 to day 8. Mice were sacrificed at day 10. Data information: values are expressed as mean ± SEM (n = 4 for each group). *P <0 .05, **P <0 .01, ***P <0 .001, ****P < 0.0001.

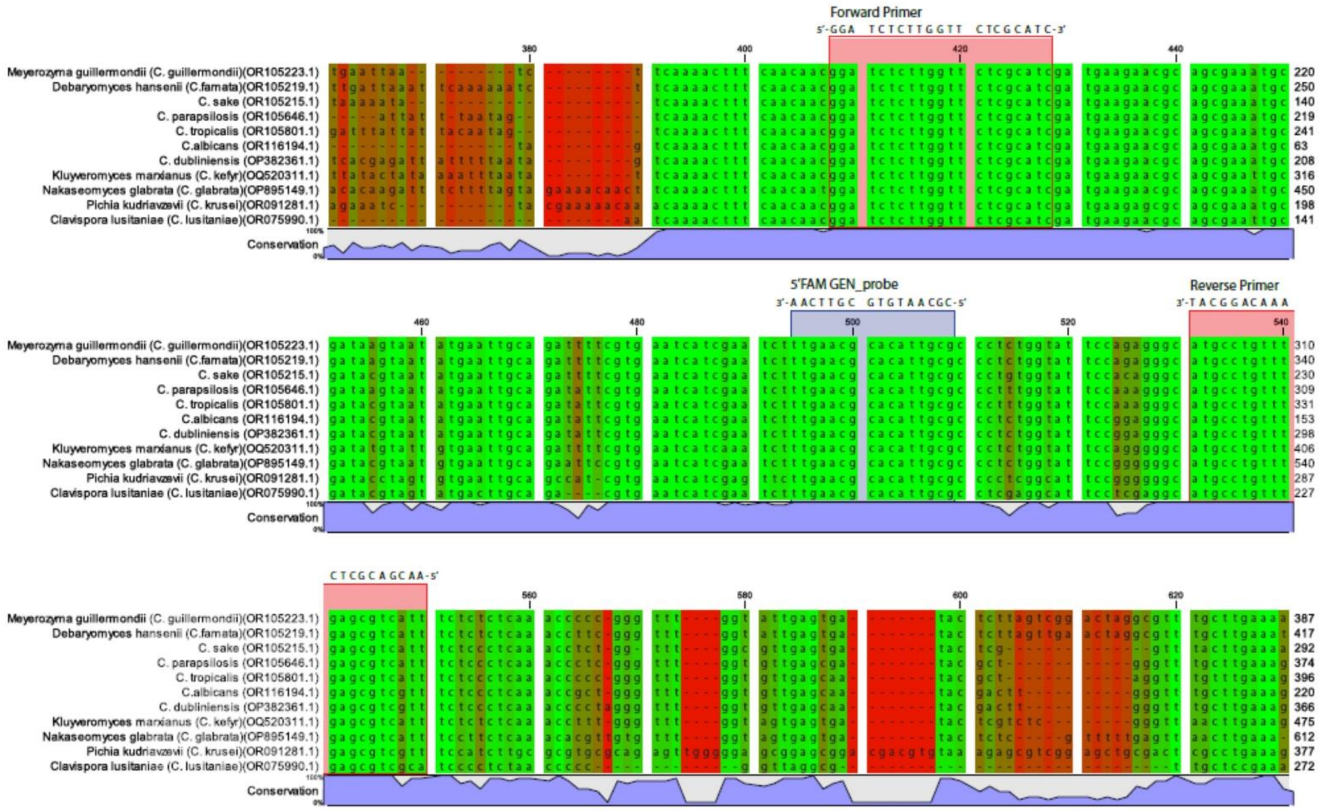

Figure S2. Sequence alignment of *Candida* spp. and the main clinically relevant former *Candida* spp. Sequence alignment was conducted using sequences retrieved from the Nucleotide database. The colors represent the level of homology across species: green indicates perfect homology, and red indicates poor homology. Additionally, the degree of conservation of the sequence between species is indicated below the alignment (represented by the blue bar). The forward and reverse primers (highlighted in red boxes) and the 5′FAM GENprobe (highlighted in the blue box) were aligned with the sequences.

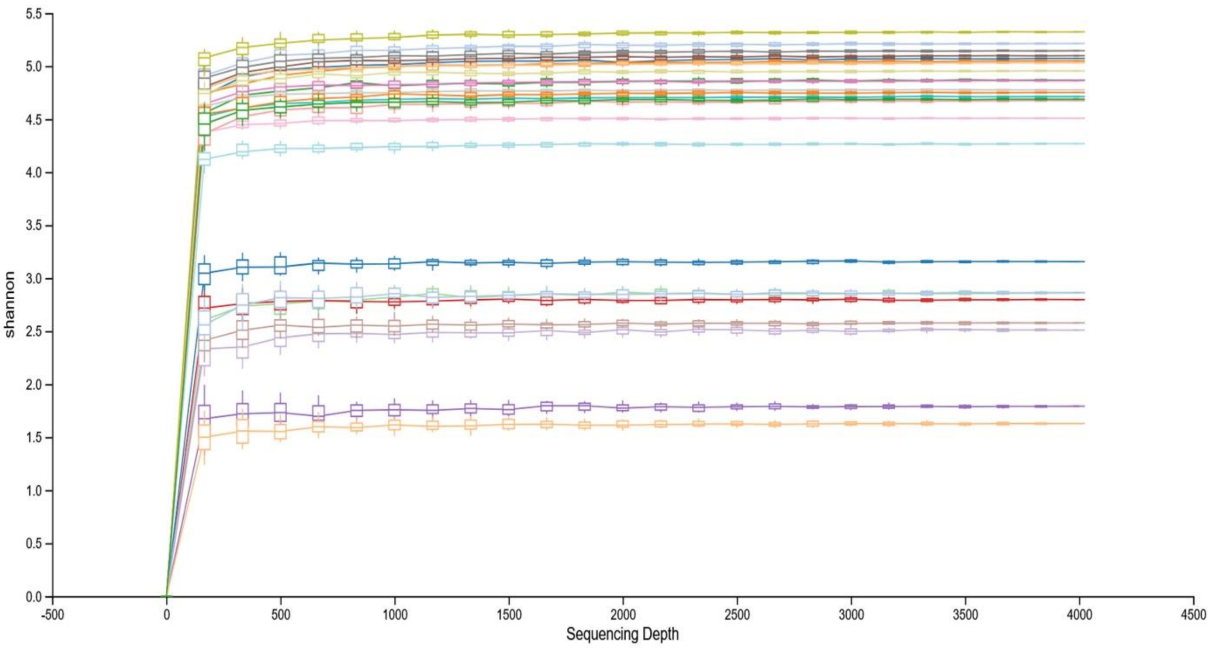

Figure S3. Rarefaction curves of the Shannon index of fecal mycobiota showing the boxplots of the index evaluated at the sequence variant level, versus sequencing depth. Each boxplot represents 50 rarefactions of each sample. The curves reach a plateau for all samples, and the treatments have distinct index values when the rarefaction is considered at 4000 reads for each sample.

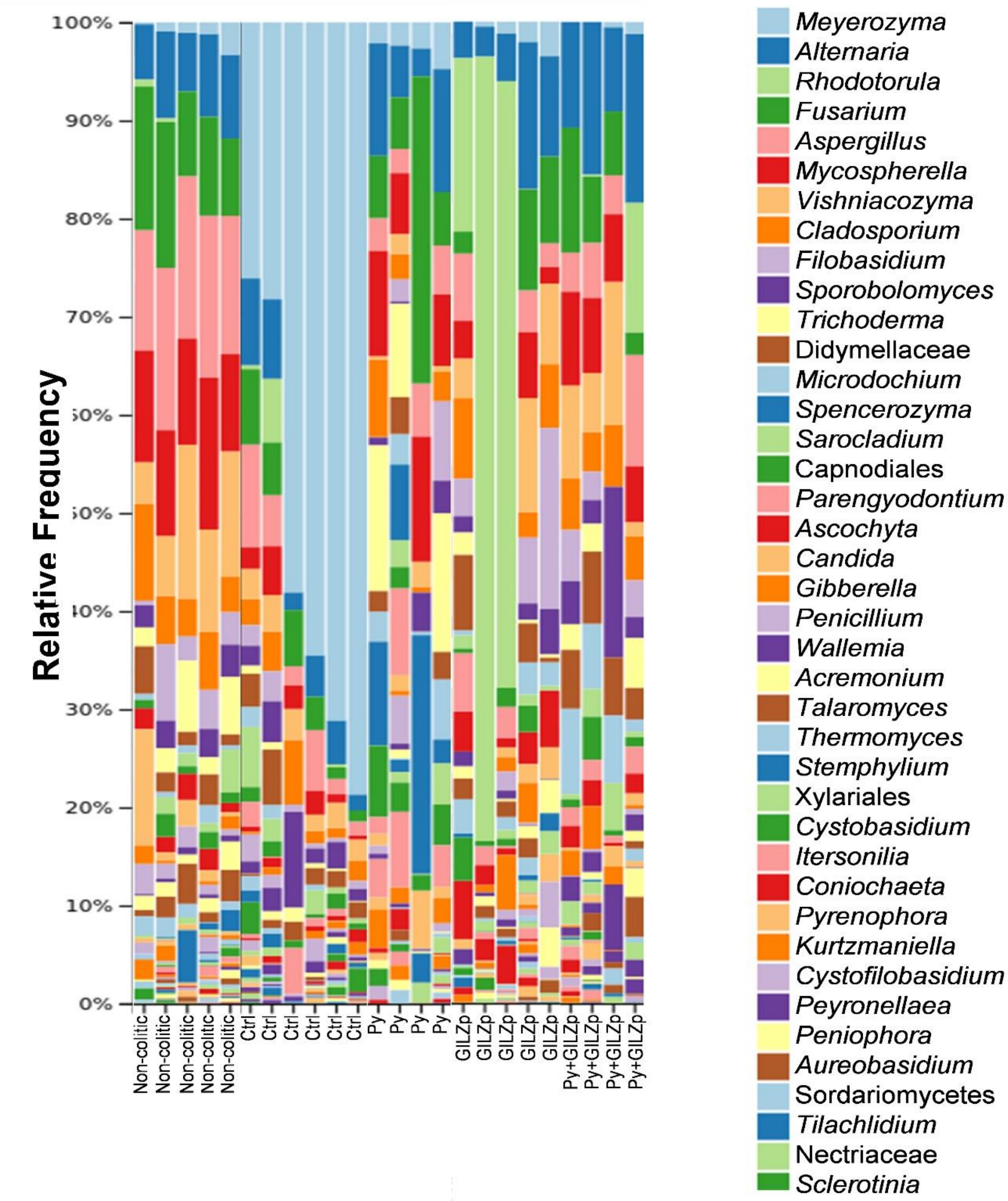

Figure S4. Mycobiome composition of the gut. Barplot of the relative abundances of genera in each group. The colors assigned to the genera indicate their abundance, and the genera are ordered from top to bottom in descending order or abundance. Within each group, samples are sorted by the most abundant genus, i.e., *Meyerozyma*.
